# Structural and Biological Properties of Water Soluble Polysaccharides from Lotus Leaves: Effects of Drying Techniques

**DOI:** 10.3390/molecules26154395

**Published:** 2021-07-21

**Authors:** Wei Li, Ding-Tao Wu, Fen Li, Ren-You Gan, Yi-Chen Hu, Liang Zou

**Affiliations:** 1 Sichuan Engineering & Technology Research Center of Coarse Cereal Industralization, Key Laboratory of Coarse Cereal Processing (Ministry of Agriculture and Rural Affairs), School of Food and Biological Engineering, Chengdu University, Chengdu 610106, China; liwei01@cdu.edu.cn (W.L.); ganrenyou@caas.cn (R.-Y.G.); huyichen0323@126.com (Y.-C.H.); 2 School of Basic Medical Sciences, Chengdu University, Chengdu 610106, China; 3 Institute of Food Processing and Safety, College of Food Science, Sichuan Agricultural University, Ya’an 625014, China; lifen19971127@163.com; 4 Research Center for Plants and Human Health, Institute of Urban Agriculture, Chinese Academy of Agricultural Sciences, Chengdu 610213, China

**Keywords:** lotus leaf, polysaccharides, drying techniques, structural properties, antioxidant, antiglycation, α-glucosidase inhibition

## Abstract

In the present study, the influence of five drying techniques on the structural and biological properties of polysaccharides from lotus leaves (LLPs) was investigated. Results revealed that the yields, contents of basic chemical components, molecular weights, and molar ratios of compositional monosaccharides of LLPs varied by different drying technologies. Low molecular weight distributions were observed in polysaccharides obtained from lotus leaves by hot air drying (LLP-H), microwave drying (LLP-M), and radio frequency drying (LLP-RF), respectively. The high contents of bound polyphenolics were measured in LLP-H and LLP-M, as well as polysaccharides obtained from lotus leaves by vacuum drying (LLP-V). Furthermore, both Fourier transform infrared (FT-IR) and nuclear magnetic resonance (NMR) spectra of LLPs were similar, indicating that drying technologies did not change their basic chemical structures. Besides, all LLPs exhibited obvious biological properties, including in vitro antioxidant capacities, antiglycation activities, and inhibitory effects on α-glucosidase. Indeed, LLP-H exhibited higher 2,2-azidobisphenol (3-ethylbenzothiazoline-6-sulfonic acid) radical scavenging ability (IC_50_ values, LLP-H, 0.176 ± 0.004 mg/mL; vitamin C, 0.043 ± 0.002 mg/mL) and 2,2-diphenyl-1-(2,4,6-trinitrate phenyl) hydrazine radical scavenging ability (IC_50_ values, LLP-H, 0.241 ± 0.007 mg/mL; butylated hydroxytoluene, 0.366 ± 0.010 mg/mL) than others, and LLP-M exerted stronger antiglycation (IC_50_ values, LLP-M, 1.023 ± 0.053 mg/mL; aminoguanidine, 1.744 ± 0.080 mg/mL) and inhibitory effects on α-glucosidase (IC_50_ values, LLP-M, 1.90 ± 0.02 μg/mL; acarbose, 724.98 ± 16.93 μg/mL) than others. These findings indicate that both hot air drying and microwave drying can be potential drying techniques for the pre-processing of lotus leaves for industrial applications.

## 1. Introduction

*Nelumbo nucifera* Gaertn. (lotus), an aquatically perennial plant, belongs to the family Nelumbonaceae, which is an edible and medicinal plant widely consumed in China [1]. Generally, lotus leaf is consumed as a popular tea material, which also has a long history, being utilized as a traditional Chinese herb for treating diverse diseases, such as haematemesis, epistaxis, hyperlipidaemia, and obesity [2]. Recently, it has also attracted increasing attention as a potential functional food due to its diverse health-promoting effects, such as antioxidant, anti-hyperglycemic, anti-proliferative, anti-inflammatory, anti-obesity, and hepatoprotective activities [1,2,3]. Usually, these health-promoting effects are correlated with different bioactive compounds that exist in lotus leaves, such as polysaccharides, flavonoids, alkaloids, essential oils, and triterpenoids [1]. Especially, polysaccharide is one of the most abundant components in the water decoction of lotus leaves, which possesses several health benefits, including obvious anti-osteoporotic, immunostimulatory, antioxidant, and antidiabetic activities [3,4,5,6,7]. Thus, polysaccharides from lotus leaves (LLPs) possess potential applications in the pharmaceutical and functional food industries.

Freshly picked lotus leaves usually contain high moisture and are thus easily spoiled, leading to the loss of beneficial ingredients. Drying can be used to quickly and effectively protect the beneficial ingredients and extend the shelf-life of medicinal and edible plants [8]. This is considered the most common and basic strategy for the pre-processing of lotus leaves. Usually, the dried lotus leaves are used as a popular tea and a traditional Chinese herb with multiple health-promoting effects in China [9]. Thus, the drying method plays a key role in the production of lotus leaf product. Generally, freeze drying (FD), vacuum drying (VD), microwave drying (MD), and hot air drying (HD), as well as radio frequency drying (RFD) are commonly used in food processing. These techniques have their own features in terms of efficiency, convenience, time, and cost. FD based on the principle of sublimation can retain the original nutrients, active ingredients, and color of postharvest plants. HD is cheap and easy to control because the postharvest plants are dried by continuous flowing hot air [10]. The plants after VD treatment can effectively reduce the structural changes because of the relatively low temperature and absence of oxygen [11]. MD has the advantages of fast drying, uniform sample energy transfer, and easy control of the drying process [12]. RFD has the advantages of uniform drying rate, short drying time, and good product quality [13]. Several studies have found that drying technologies have an important effect on the chemical properties and biological activities of plant polysaccharides [14,15,16]. A previous study revealed that oven drying (55–60 °C) and microwave drying (680–850 W) can affect the quality and antioxidant activity of lotus leaves [17].

However, whether the drying technologies can affect the chemical properties, such as molecular weight, compositional monosaccharides, and chemical components of polysaccharides from lotus leaves remains unknown. Indeed, the effect of drying technologies on the antioxidant and antidiabetic activities of polysaccharides from lotus leaves is also unknown. Therefore, different drying techniques were applied for the pre-processing of lotus leaves in this study, and the effects of drying techniques on the physicochemical (basic chemical components, molecular weight distribution, monosaccharide composition, and chemical structure) and biological properties (antioxidant capacities, antiglycation activities, and inhibitory effects on α-glucosidase) of LLPs were carefully evaluated.

## 2. Materials and Methods

### 2.1. Materials and Chemicals

Lotus leaves (*Nelumbo nucifera* cv. Elian 6) were collected from a lotus planting base (Renshou Rennong Lotus Professional Cooperative, Renshou, Sichuan, China) on 28 May 2019 before the blooming of lotus. Then, lotus leaves were washed and cut into about 4 square centimeters before drying.

Monosaccharide standards, aminoguanidine, 1-phenyl-3-methyl-5-pyrazolinone (PMP), trifluoroacetic acid (TFA), 2,2-azidobisphenol (3-ethylbenzothiazoline-6-sulfonic acid) (ABTS), m-hydroxybiphenyl, 2,2-diphenyl-1-(2,4,6-trinitrate phenyl) hydrazine (DPPH), Griess reagent, acarbose, α-glucosidase from *Bacillus stearothermophilus*, and 4-nitrophenyl β-D-pyran glucoside were obtained from Sigma-Aldrich. All other chemicals and solvents were of analytical grade.

### 2.2. Drying Processes

The RFD, HD, FD, MD, and VD techniques were applied for the pre-processing of lotus leaves. The RFD was performed by using a pilot-scale 8 kW, 27.12 MHz RF heating system (Shijiazhuang Huas Jiyuan High Frequency Equipment Co., Ltd., Shijiazhuang, China) at the power of 700 W and temperature of 60 °C for 1.5 h according to the procedure described by a previous study [18]. Other drying methods were also carried out according to a previous study [15]. Briefly, the HD was carried out with hot air at 75 °C for 3.5 h (101A-3, Shanghai Experimental Instrument Factory Co., Ltd., Shanghai, China). For the FD, lotus leaves were frozen at −80 °C and then dried using a freeze dryer for 48 h (SCIENTZ-12N, Ningbo Scientz Biotechnology Co., Ltd., Ningbo, China). The VD was conducted at 50 °C for 21 h (DZF-6050, Shanghai San Fa Scientific Instruments Factory Co., Ltd., Shanghai, China). The MD was performed at 85 °C and 400 W for 13 min (MKJ-J1-3, Qingdao Makewave Microwave Applied Technology Co., Ltd., Shandong, China). The drying experiments lasted until the moisture contents of lotus leaves were less than approximately 10% (wet basis) as detected by a moisture meter. After drying, the lotus leaves were stored at −20 °C before hot water extraction.

### 2.3. Preparation of LLPs

Polysaccharides in lotus leaves were extracted with hot water, as previously reported [15]. Briefly, the powder of dried lotus leaves (10.0 g) was pre-treated with 80% methanol (*v*/*v*) twice for 1 h to remove small molecules, and then the residues were extracted twice with deionized water (300.0 mL) at 90 °C for 3 h. The extract was concentrated, precipitated with 95% ethanol (*v*/*v*), dialyzed (molar mass cutoff: 3.0 kDa), and lyophilized to obtain polysaccharides from lotus leaves dried by HD, MD, VD, RFD, and FD, which were then encoded as LLP-H, LLP-M, LLP-V, LLP-RF, and LLP-F, respectively.

### 2.4. Characterization of Physicochemical Properties of LLPs

#### 2.4.1. Chemical Components of LLPs

The contents of total polysaccharides, the contents of uronic acids, the contents of proteins, and the contents of bound polyphenolics in LLPs obtained by different drying technologies were detected based on the previous studies [19,20].

#### 2.4.2. Determination of Molecular Weights and Monosaccharide Compositions of LLPs

The molecular weights (*M_w_*) of LLPs were measured by size-exclusion chromatography followed by multi-angle laser light scattering detection and refractive index detection (SEC-MALLS-RID, Wyatt Technologies, Santa Barbara, CA, USA) as previously reported [15]. The Shodex OHpak SB-806M HQ column was applied to separate LLPs at a stable temperature of 30 °C. The monosaccharide compositions of LLPs obtained by different drying technologies were detected on a Thermo U3000 HPLC system (ThermoFisher, Waltham, MA, USA) coupled with a phenomenex gemini C18 110A column as previously reported [20].

#### 2.4.3. Analysis of FT-IR Spectra of LLPs

FT-IR spectra of LLPs were recorded on a Nicolet iS 10 FT-IR (ThermoFisher scientific, Waltham, MA, USA) in the frequency range of 4500–400 cm^−1^ based on a previous study [15]. The DE value was determined according to the band areas of 1700–1750 cm^−1^ and 1600–1630 cm^−1^, and calculated by the following formula,

(1) $$\mathrm{DE}\;\left(\%\right)=\left(\frac{A_{1734}}{A_{1734}+{\;A}_{1616}}\right)\times 100$$

#### 2.4.4. Analysis of NMR Spectra of LLPs

The ^1^H and ^13^C NMR spectra of LLPs obtained by different drying technologies were tested by using a Bruker Ascend 600 MHz spectrometer with a z-gradient probe (Bruker, Rheinstetten, Germany). The frequencies of carbon and proton were 150.90 and 600.13 MHz, respectively. The acquisition temperature, internal reference, and number of scans were 298 k, 3-(trimethylsilyl) propionic-2,2,3,3-d4 acid sodium salt, and 6000, respectively.

### 2.5. Determination of In Vitro Antioxidant Activities, Antiglycation Activities, and α-Glucosidase Inhibitory Effects of LLPs

The in vitro antioxidant activities of LLPs obtained by different drying technologies were measured by the ABTS and DPPH radical scavenging assays, as well as the ferric reducing antioxidant power (FRAP) assay according to a previous study [16]. The IC_50_ values (mg/mL) of radical scavenging abilities were determined by a logarithmic regression curve, while the absorbance of the reaction solution at 593 nm represented the FRAP of LLPs.

The antiglycation activities of LLPs obtained by different drying technologies were determined by a BSA-glucose model, which was also conducted based on a previous study with slight modifications [14]. The samples were measured at the concentrations ranged from 0.25–4.00 mg/mL, and aminoguanidine (AG) was used as the positive control.

The α-glycosidase inhibitory activities of LLPs were also tested based on a reported study [21]. LLP-H, LLP-M, LLP-V, and LLP-RF were determined at concentrations ranging from 2.0 to 6.0 μg/mL, respectively. Besides, LLP-F was measured at concentrations ranging from 5 to 25 μg/mL. Acarbose was used as the positive control in this study.

### 2.6. Statistical Analysis

All experiments in this study were performed in triplicate, while the data were expressed in means ± standard deviations. Statistical significances were carried out by analysis of variance (ANOVA) plus post hoc Duncan’s test using SPSS software.

## 3. Results and Discussion

### 3.1. Physicochemical Properties of LLPs Affected by Different Drying Techniques

#### 3.1.1. Basic Chemical Components of LLPs

The contents of moisture in lotus leaves after HD, MD, VD, RFD, and FD treatments were measured to be 6.54% ± 0.24%, 7.66% ± 0.16%, 7.57% ± 0.15%, 8.07% ± 0.17%, and 6.91% ± 0.25%, respectively. The yields and basic chemical components of LLPs prepared by different drying techniques are presented in Table 1. The yields of LLPs were influenced by drying technologies, ranging from 3.44% ± 0.55% to 4.57% ± 0.64%, which were higher compared to the results (0.97–1.93%) of a previous study [6]. The yields of LLP-RF and LLP-F were higher than that of other tested LLPs. It is reported that freeze drying causes more porous microstructures and ice crystals to form cellular structures in the plant matrix to promote solvent penetration during the extraction process [22]. The yields also confirmed that the radio frequency drying technique was a valuable drying process for obtaining polysaccharides from lotus leaves. The contents of polysaccharides and proteins in LLPs were also influenced by drying technologies, which ranged from 66.43% ± 1.57% to 76.35% ± 1.17%, and from 4.44% ± 0.50% to 8.76% ± 0.67%, respectively. Results revealed that the main components in LLPs were polysaccharides. Furthermore, the contents of uronic acids in LLPs were also influenced by drying technologies, ranging from 12.92% ± 0.23% to 20.29% ± 1.74%. The highest uronic acid content was tested in LLP-F among LLPs, which might be due to the lowest oxygen concentration and the lowest temperature during freeze drying [16]. The high content of uronic acids in LLPs indicated the presence of pectic-polysaccharides in lotus leaves [14]. Moreover, some polyphenolics were still found in LLPs after methanol extraction, ethanol precipitation, and dialysis treatments. The contents of bound polyphenolics in LLPs ranged from 47.36 ± 2.70 mg GAE/g to 119.87 ± 2.76 mg GAE/g, which were also significantly influenced by drying technologies. The long heat treatment could reduce the porosity of samples and increase the apparent saturation and apparent affinity. Moreover, the long heat treatment could also destroy the physical structure of the sample matrix, making it easier for small molecules to combine with polysaccharides [23]. Therefore, the contents of bound polyphenolics in LLP-H, LLP-V, and LLP-M were higher than that of others. Generally, the bound polyphenolics can enhance the biological activities of natural polysaccharides [19,24].

#### 3.1.2. Molecular Weight Distributions and Monosaccharide Compositions of LLPs

**Figure 1:**
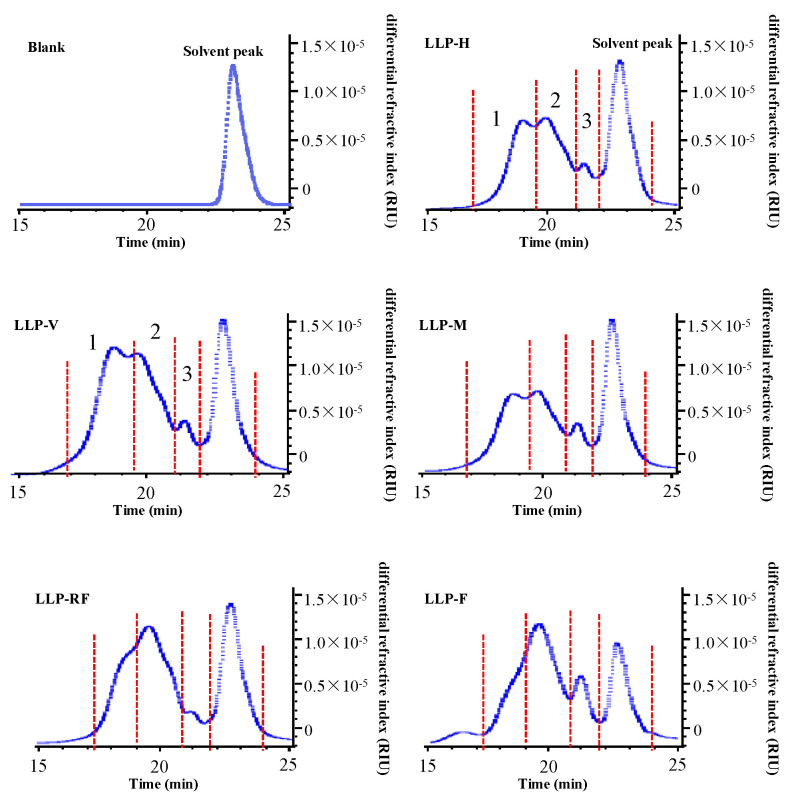
High-performance size-exclusion chromatograms of LLPs.

According to previous studies, the biological functions of polysaccharides are usually associated with their compositional monosaccharides and molecular weights [20]. Thus, we compared the monosaccharides and molecular weight distributions of LLPs. Figure 1 shows that HPSEC-RID chromatograms of LLP-H, LLP-M, and LLP-V were similar, but LLP-RF and LLP-F were slightly different from them. Three distinct fractions were found in each sample, which characterized as polysaccharide fraction 1 (0.81–1.94 × 10^5^ Da), polysaccharide fraction 2 (1.12–7.41 × 10^4^ Da), and polysaccharide fraction 3 (5.17–60.09 × 10^3^ Da). Results indicated that molecular weight distributions of LLPs varied by drying technologies. The detailed molecular weight distributions of polysaccharide fractions 1–3 in LLPs are summarized in Table 2 and are similar to the results of a previous study [4]. Molecular weights of LLP-H, LLP-M, and LLP-RF were obviously lower than that of LLP-F and LLP-V, because the increase of temperature might cause the decrease of molecular weights [22]. It is observed that polysaccharides with low molecular weight possess much higher antioxidant activities than that of polysaccharides with high molecular weight [25,26]. Furthermore, the polydispersities of fraction 1 ranged from 1.10 to 1.72, fraction 2 ranged from 1.06 to 1.42, and fraction 3 ranged from 1.01 to 1.05, consistent with HPSEC chromatograms.

**Figure 2:**
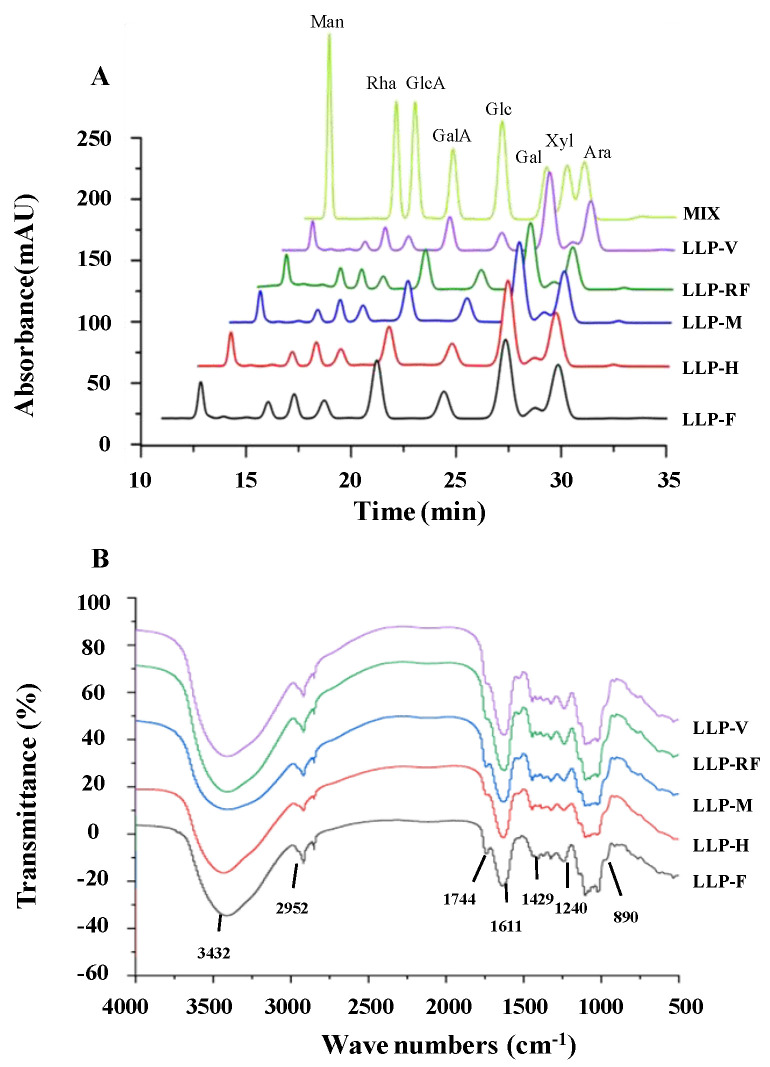
HPLC profiles of constituent monosaccharides (**A**) and FT-IR spectra (**B**) of LLPs.

In addition, Figure 2A shows that all LLPs were mainly composed of galactose (Gal), arabinose (Ara), galacturonic acid (GalA), rhamnose (Rha), glucose (Glc), mannose (Man), and glucuronic acid (GlcA), as well as minor xylose (Xyl). However, the molar ratios of LLPs varied by drying technologies (Table 2). The high molar ratio of GalA was observed in LLP-F, which was in accordance with the content of uronic acids (Table 1). Based on the molar ratios, Gal, GalA, and Ara were identified as the dominant monosaccharides in LLPs, consistent with a previous study [6]. Results further indicated that pectin-type polysaccharides existed in LLPs [15,16].

The sample codes were as the same in Table 1.

#### 3.1.3. FT-IR and NMR Spectra of LLPs

FT-IR analysis showed that LLPs obtained by different drying technologies had similar IR absorption profiles (Figure 2B), indicating that LLPs possessed similar chemical groups. The strong absorption bands at the ranges of 3600–3000 cm^−1^, 3000–2800 cm^−1^, 1700–1500 cm^−1^, 1400–1200 cm^−1^, and 1200–700 cm^−1^ were typical bands of polysaccharides [4,6]. The absorption band at 3432 cm^−1^ was responsible for the stretching vibration of -OH group [6], and the tensile vibration at 2952 cm^−1^ was assigned to the signal of C-H [14]. The absorption band at 1744 cm^−1^ suggested the stretching of the esterified carboxyl group, while the relatively strong absorption band at about 1611 cm^−1^ indicated the existence of carboxyl or carbonyl groups in LLPs, further confirming that LLPs contained acidic polysaccharides [16]. The absorption band at 1429 cm^−1^ was the feature of the C-H or the O-H [15]. Moreover, the absorption band around 1240 cm^−1^ could be caused by the C-O-C of the methyl ester groups. In addition, absorption bands between 1200 cm^−1^ and 1000 cm^−1^ showed stretching vibrations of the C-O-H and the C-O-C, suggesting that the pyranose sugars existed in LLPs [6]. Moreover, the characteristic absorption band at 890 cm^−1^ indicated the β-glycosidic linkages in LLPs [27]. Furthermore, the degrees of esterification (DE) of LLPs obtained by different drying technologies were also determined by FT-IR spectroscopy analysis. As shown in Table 1, the DE values of LLPs ranged from 13.34% to 22.34%. The highest DE value (22.34%) was found in LLP-F among LLPs, and the lowest DE value (13.34%) was found in LLP-H. Previous studies revealed that the lower DE value of natural polysaccharides might be closely associated with their higher antioxidant activity [16].

The sample codes were as presented in Table 1.

**Figure 3:**
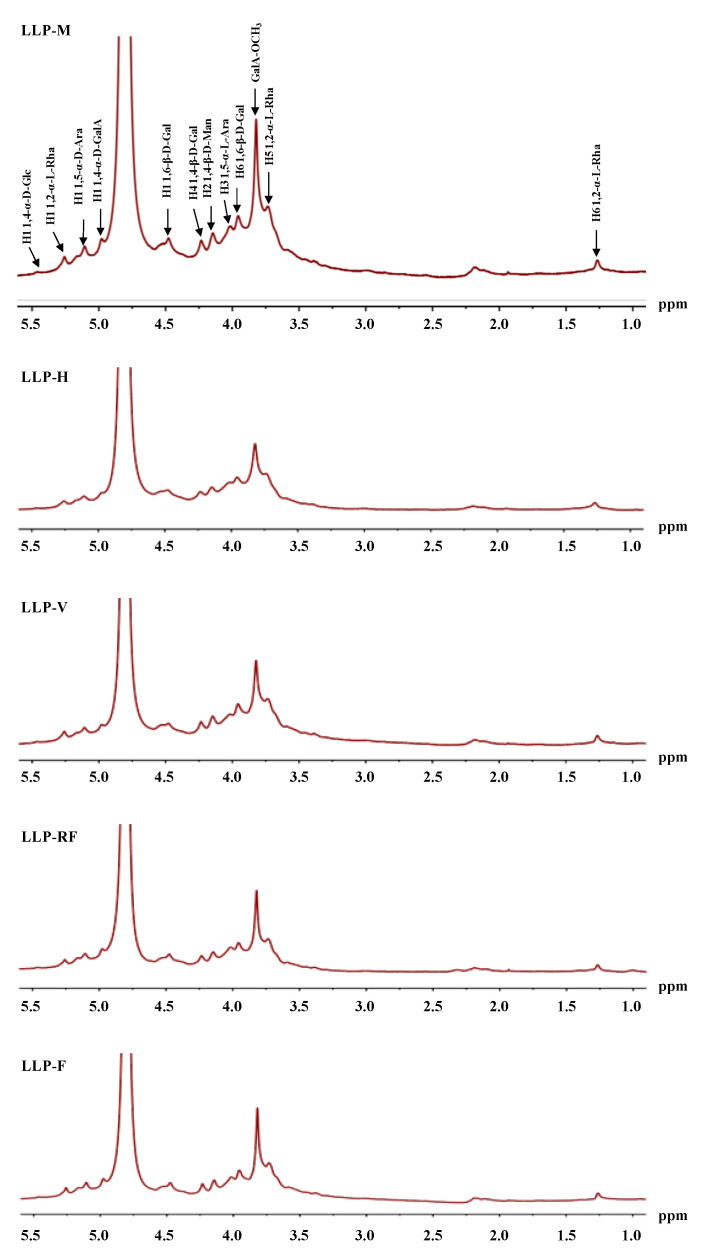
^1^H NMR spectra of LLPs.

**Figure 4:**
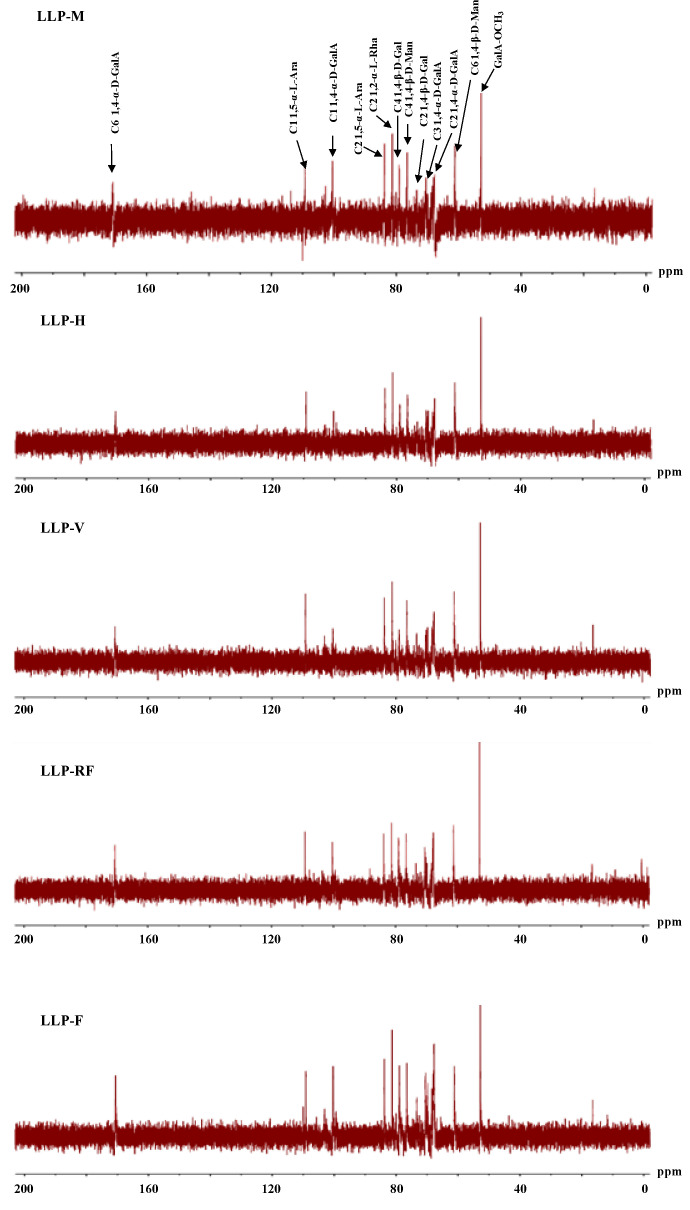
^13^C NMR spectra of LLPs.

In order to reveal the detailed structural features of LLPs obtained by different drying techniques, the NMR spectra were also recorded to analyze their structural information. As shown in Figure 3 and Figure 4, the ^1^H and ^13^C NMR spectra of LLPs obtained by different drying technologies were similar, indicating that drying technologies did not change their backbones (main glycosidic linkages). The ^1^H signal at 4.80 ppm was assigned to HOD. The signal at 4.98 ppm in the ^1^H NMR spectra was assigned to the H-1 of 1, 4-α-D-GalA linkage, and its signals of C-1, C-2, C-3, and C-6 were 100.39 ppm, 68.07 ppm, 70.80 ppm, and 170.61 ppm, respectively [18,28]. A very intense signal at 3.82 ppm could be assigned to the methyl ester groups of the GalA carboxyl groups with the signal at 52.81 ppm in the ^13^C NMR spectra [18]. Signals at 5.26, 3.73, and 1.26 ppm were attributed to the H-1, H-5, and H-6 of 1, 2-α-L-Rha linkage, respectively, and 81.29 ppm was assigned to the C-2 of 1, 2-α-L-Rha linkage [29]. The signal at 4.48 and 3.96 ppm belonged to the H-1 and H-6 of 1, 6-β-D-Gal linkage [18,30]. The signal at 4.23 ppm was assigned to the H-4 of 1, 4-β-D-Gal linkage, and the peaks at 73.18 and 78.93 ppm were connected to the C-2 and C-4 of 1, 4-β-D-Gal linkage [18,31]. The peaks at 5.11 and 4.02 ppm suggested the H-1 and H-3 of 1, 5-α-L-Ara linkage, respectively, with signals at 109.16 and 83.79 ppm were derived from the C-1 and C-2 of 1, 5-α-L-Ara linkage [32], respectively. The signal at 4.16 ppm was derived from the H-2 of 1, 4-β-D-Man linkage, while 76.74 and 60.77 ppm were corresponded to the C-4 and C-6 of 1, 4-β-D-Man linkage [33]. The signals at 5.46 corresponded to the H-1 of 1,4-α-D-Glc linkage [18]. Finally, results suggested that rhamnogalacturonan I (RG I), homogalacturonan (HG), and arabionogalactan might exist in LLPs according to their monosaccharides, FT-IR, and NMR spectra [34].

The sample codes were as the same in Table 1.

The sample codes were as the same in Table 1.

### 3.2. In Vitro Antioxidant Activities of LLPs Affected by Different Drying Techniques

**Figure 5:**
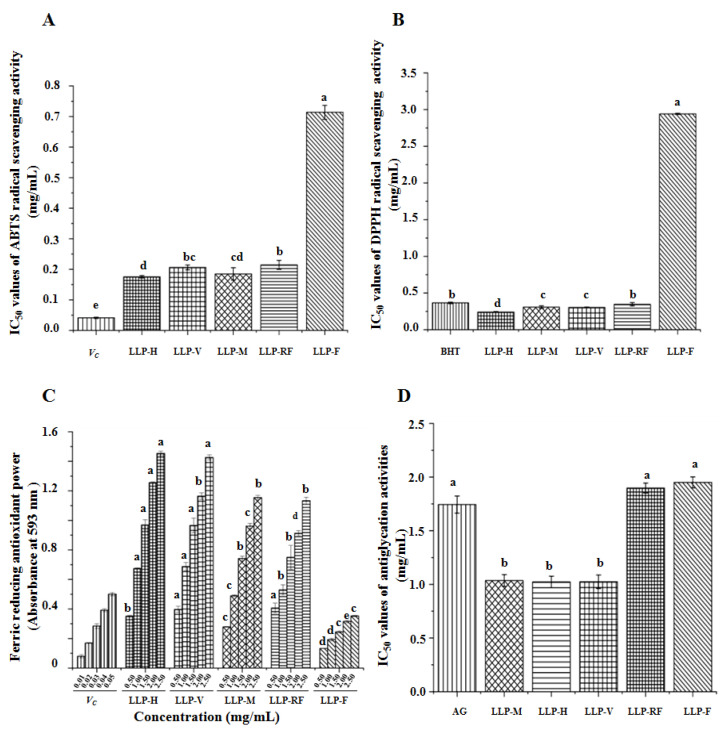

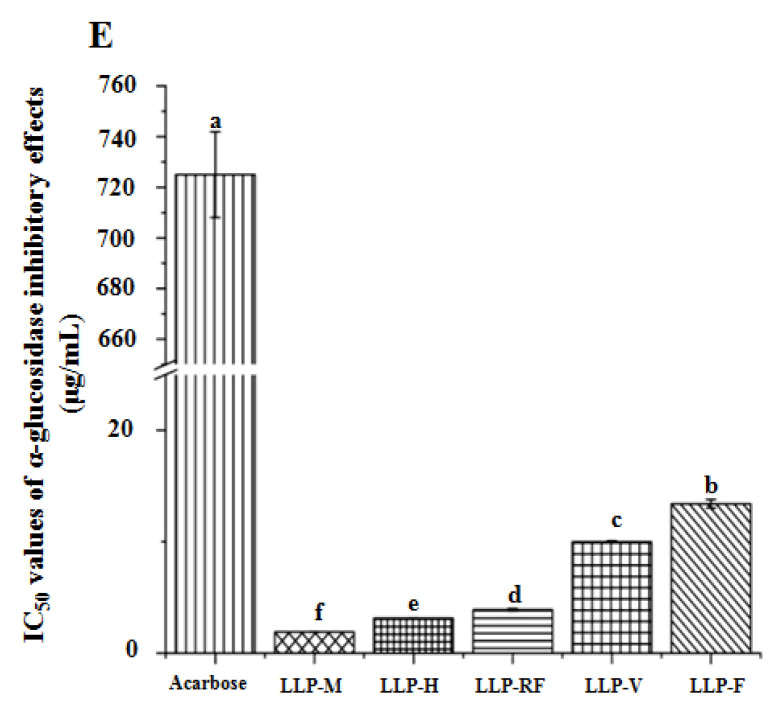
ABTS (**A**), DPPH (**B**), and FRAP (**C**) antioxidant capacities, antiglycation activities (**D**), and inhibitory effects on α-glucosidase (**E**) of LLPs.

In addition to the physicochemical properties, the biological properties of LLPs were further evaluated and compared. Several studies revealed that polysaccharides extracted from lotus leaves possessed significant antioxidant activities [4,6]. In this study, we compared the ABTS and DPPH radical scavenging activities, as well as the FRAP values of LLPs prepared by different drying techniques. As shown in Figure 5, LLPs obtained by different drying technologies exhibited potential antioxidant activities compared to the positive controls. Indeed, results indicated that antioxidant activities of LLPs varied by different drying technologies, which might be related to the different chemical properties affected by drying technologies. Briefly, the IC_50_ values of ABTS and DPPH radical scavenging activities of LLPs obtained by different drying techniques ranged from 0.176 mg/mL to 0.714 mg/mL, and from 0.241 mg/mL to 2.942 mg/mL, respectively. Moreover, the FRAP values of LLPs obtained by different drying techniques ranged from 0.353 ± 0.004 to 1.454 ± 0.015 at the concentration of 2.5 mg/mL. Results indicated that LLP-H obtained by hot air drying exhibited higher ABST, DPPH, and FRAP antioxidant capacities than that of others, suggesting that the hot air drying technique might be a good method for drying lotus leaves to obtain polysaccharides with high antioxidant activities. In addition, compared with LLP-F, LLP-M, LLP-V, and LLP-RF also exhibited stronger antioxidant capacities.

Generally, a number of studies have demonstrated that the antioxidant activities of plant polysaccharides are connected to the chemical characteristics, molecular weight distributions, compositional monosaccharides (uronic acids), contents of proteins, degree of esterification, and bound polyphenolics [14,16,35]. Polysaccharides with lower molecular weight distributions exhibit higher antioxidant activities [21,22], which may be due to their more loose and porous structures, resulting in more reductive hydroxyl groups to react with free radicals. Indeed, the contents of free uronic acids can also contribute to the antioxidant activities of polysaccharides due to the electrophilic groups of acidic polysaccharides, which can facilitate the liberation of hydrogens. Moreover, the low DE value of natural polysaccharides could contribute to their relatively high antioxidant activity [16]. Furthermore, the bound polyphenolics can also enhance the biological activities of natural polysaccharides [19,24]. In the present study, compared with LLP-F, the higher antioxidant capacities observed in LLP-H, LLP-M, LLP-V, and LLP-RF might be due to the combination effect of low molecular weight, high content of free uronic acids, and high content of bound polyphenolics [14,18,21]. However, further studies are required to reveal the detailed structure-antioxidant activity of LLPs in the future.

The sample codes were as the same in Table 1. BHT, butylated hydroxytoluene; Vc, vitamin C; AG, aminoguanidine; The error bars are standard deviations; Significant (*p* < 0.05) differences are shown by data bearing different letters (a–e); Statistical significances were carried out by ANOVA and post hoc Duncan’s test.

### 3.3. In Vitro Antiglycation Activities of LLPs Affected by Different Drying Techniques

Advanced glycosylation end products (AGEs) can lead to several chronic diseases, such as arteriosclerosis, aging, and diabetes complications, which are produced by the spontaneous non-enzymatic aminocarbonyl reaction between reducing sugars and proteins [14]. In this study, we compared the antiglycation activities of LLPs obtained by different drying technologies. Figure 5D shows that LLPs possessed stronger antiglycation activities than that of AG (IC_50_ = 1.744 ± 0.080 mg/mL). The antiglycation activities of LLPs also varied by different drying technologies, and their IC_50_ values ranged from 1.023 to 1.951 mg/mL. Results revealed that LLP-M, LLP-H, and LLP-V exerted stronger antiglycation activities than LLP-RF and LLP-F. Previous studies suggested that the antiglycation effect might be associated with the antioxidant activity [14,36], and the high content of bound polyphenolics also contributed to their antiglycation effect.

### 3.4. In Vitro α-Glucosidase Inhibitory Effects of LLPs Affected by Different Drying Techniques

The α-glucosidase inhibitory activity can combat the metabolic changes associated with type 2 diabetes, and it is also one of the major strategies for treatment of type 2 diabetes [37]. Some studies revealed that pectin-type polysaccharides possessed anti-diabetic activities [21,38], and polysaccharides from lotus leaves also exerted anti-diabetic effect [7]. Therefore, we compared the in vitro α-glucosidase inhibitory activities of LLPs obtained by different drying technologies (Figure 5E). Potent inhibitory effects against α-glucosidase of LLPs were observed, which also varied by different drying technologies. The IC_50_ values of LLPs ranged from 1.90 ± 0.02 to 13.40 ± 0.38 μg/mL, which were significantly lower than that of acarbose (IC_50_ = 724.98 ± 16.93 µg/mL). The results revealed that LLP-M prepared by microwave drying exhibited the highest α-glucosidase inhibitory activity among LLPs, while the weakest α-glucosidase inhibitory effect was observed in LLP-F among LLPs. In addition, the α-glucosidase inhibitory of LLPs was higher than that of polysaccharides obtained from dandelion [18] and okra [16].

Generally, polysaccharides can bind with the free α-glucosidase to change its enzyme structure, decreasing its catalytic abilities [39,40]. At the same time, polysaccharides can also restrain the digestion of substrate by binding to the substrate and enzyme to form a ternary complex [39]. Usually, the inhibitory effects of natural polysaccharides on α-glucosidase are associated with their chemical properties, such as molecular weights, monosaccharide compositions, contents of free uronic acids, and contents of bound polyphenolics. Studies have reported that polysaccharides with lower molecular weights from *Zingiber* *officinale* and blackberry fruit exhibit higher inhibitory activities on digestive enzymes [39,40]. Additionally, the high degree of esterification of pectic-polysaccharides might contribute to their high inhibitory effects on digestive enzymes [41]. Furthermore, the high content of bound polyphenolics may also contribute to the high inhibitory effects of natural polysaccharides against digestive enzymes [14,18,42]. Therefore, in the present study, the lower molecular weight distribution, higher degree of esterification, and higher content of bound polyphenolics of LLP-M might contribute to its stronger inhibitory effect on α-glucosidase. However, besides the chemical components determined in LLPs (Table 1), other phytochemicals might also exist in LLPs. Therefore, further studies are required to well reveal the potential structure–function relationships of LLPs in the future.

## 4. Conclusions

In this study, the physicochemical and biological properties of polysaccharides from lotus leaves obtained by different drying technologies were systematically evaluated. The results revealed that drying techniques could alter the physicochemical characteristics and biological activities of lotus leave polysaccharides. The low molecular weight distributions were observed in LLP-H, LLP-M, and LLP-RF, respectively. The high contents of bound polyphenolics were measured in LLP-H, LLP-M, and LLP-V, respectively. Besides, all LLPs exhibited obvious in vitro antioxidant capacities, antiglycation activities, and inhibitory effects on α-glucosidase. LLP-H exhibited significant higher antioxidant activities than others, and LLP-M exerted stronger antiglycation and inhibitory effects on α-glucosidase than others. The findings from this study could provide fundamental knowledge for selecting suitable drying methods for the pre-processing of lotus leaves.

## Figures and Tables

**Table 1:** Extraction yields and chemical compositions of LLPs.

	LLP-H	LLP-M	LLP-V	LLP-RF	LLP-F
Extraction yields (%)	3.44 ± 0.55 ^b^	3.84 ± 0.59 ^ab^	3.70 ± 0.65 ^ab^	4.57 ± 0.64 ^a^	4.17 ± 0.99 ^ab^
Total polysaccharides (%)	72.05 ± 2.27 ^b^	68.49 ± 1.04 ^c^	76.35 ± 1.17 ^a^	72.01 ± 1.98 ^b^	66.43 ± 1.57 ^c^
Total uronic acids (%)	14.65 ± 0.66 ^b^	12.92 ± 0.23 ^c^	13.29 ± 1.05 ^bc^	13.84 ± 0.40 ^bc^	20.29 ± 1.74 ^a^
Total polyphenolics (mg GAE/g)	119.87 ± 2.76 ^a^	109.67 ± 1.22 ^b^	113.37 ± 2.37 ^b^	81.55 ± 2.87 ^c^	47.36 ± 2.70 ^d^
Total proteins (%)	8.76 ± 0.67 ^a^	8.17 ± 0.36 ^a^	8.60 ± 0.25 ^a^	6.76 ± 0.36 ^b^	4.44 ± 0.50 ^c^
Degree of esterification (%)	13.34 ± 0.20 ^c^	20.43 ± 0.57 ^ab^	16.79 ± 0.24 ^bc^	14.60 ± 0.57 ^c^	22.34 ± 0.20 ^a^

LLP-H, LLP-M, LLP-V, LLP-RF and LLP-F are polysaccharides from lotus leaves obtained by hot air drying, microwave drying, vacuum drying, radio frequency drying, and freeze drying, respectively; Values represent mean ± standard deviation, and superscripts ^a–d^ differ significantly (*p* < 0.05) among LLPs; Statistical significances were carried out by ANOVA plus post hoc Ducan’s test.

**Table 2:** Molecular weight (*M_w_*) and constituent monosaccharides of LLPs.

	LLP-H	LLP-M	LLP-V	LLP-RF	LLP-F
*M_w_* (Da)					
Fraction 1 (×10^5^)	0.83 (±0.40%)	0.81 (±0.32%)	1.45 (±0.28%)	0.83 (±0.28%)	1.94 (±0.97%)
Fraction 2 (×10^4^)	1.12 (±1.69%)	1.67 (±1.31%)	2.15 (±0.62%)	1.49 (±0.89%)	7.41 (±1.25%)
Fraction 3 (×10^3^)	5.17 (±4.02%)	7.92 (±4.71%)	18.21 (±1.72%)	6.87 (±7.16%)	60.09 (±1.85%)
Monosaccharides and molar ratios					
Galactose	1.00	1.00	1.00	1.00	1.00
Galacturonic acid	0.69	0.69	0.60	0.82	1.02
Arabinose	0.74	0.78	0.68	0.76	0.82
Rhamnose	0.30	0.28	0.30	0.30	0.31
Glucose	0.33	0.31	0.25	0.32	0.37
Mannose	0.25	0.25	0.24	0.49	0.30
Glucuronic acid	0.19	0.17	0.17	0.16	0.20
Xylose	0.03	0.04	0.11	0.03	0.04

The sample codes were as the same in Table 1; Values represent mean ± standard deviation, and superscripts a-d differ significantly (*p* < 0.05) among LLPs; Statistical significances were carried out by ANOVA plus post hoc Ducan’s test.

## Data Availability

Not applicable.

## References

[1] Chen G., Zhu M., Guo M. (2019). Research advances in traditional and modern use of *Nelumbo nucifera*: Phytochemicals, health promoting activities and beyond. Crit. Rev. Food Sci. Nutr..

[2] Mukherjee P.K., Mukherjee D., Maji A.K., Rai S., Heinrich M. (2009). The sacred lotus (*Nelumbo nucifera*)—phytochemical and therapeutic profile. J. Pharm. Pharmacol..

[3] Hwang Y.-H., Jang S.-A., Lee A., Cho C.-W., Song Y.-R., Hong H.-D., Ha H., Kim T. (2020). Polysaccharides isolated from lotus leaves (LLEP) exert anti-osteoporotic effects by inhibiting osteoclastogenesis. Int. J. Biol. Macromol..

[4] Zhang L., Tu Z.C., Wang H., Kou Y., Wen Q.H., Fu Z.F., Chang H.X. (2015). Response surface optimization and physicochemical properties of polysaccharides from *Nelumbo nucifera* leaves. Int. J. Biol. Macromol..

[5] Song Y.-R., Han A.-R., Lim T.-G., Lee E.-J., Hong H.-D. (2019). Isolation, purification, and characterization of novel polysaccharides from lotus (*Nelumbo nucifera*) leaves and their immunostimulatory effects. Int. J. Biol. Macromol..

[6] Song Y.-R., Han A.-R., Park S.-G., Cho C.-W., Rhee Y.-K., Hong H.-D. (2020). Effect of enzyme-assisted extraction on the physicochemical properties and bioactive potential of lotus leaf polysaccharides. Int. J. Biol. Macromol..

[7] Zeng Z.H., Xu Y., Zhang B. (2017). Antidiabetic activity of a lotus leaf selenium (Se)-polysaccharide in rats with gestational diabetes mellitus. Biol. Trace Elem. Res..

[8] Babu A.K., Kumaresan G., Raj V.A.A., Velraj R. (2018). Review of leaf drying: Mechanism and influencing parameters, drying methods, nutrient preservation, and mathematical models. Renew. Sust. Energ. Rev..

[9] Huang B., Ban X., He J., Tong J., Tian J., Wang Y. (2010). Hepatoprotective and antioxidant activity of ethanolic extracts of edible lotus (*Nelumbo nucifera* Gaertn.) leaves. Food Chem..

[10] Li H.G., Xia N., Hasselwander S., Daiber A. (2019). Resveratrol and vascular function. Int. J. Mol. Sci..

[11] Wang Y., Li X., Zhao P., Qu Z., Bai D., Gao X., Zhao C., Chen J., Gao W. (2019). Physicochemical characterizations of polysaccharides from *Angelica*
*sinensis* Radix under different drying methods for various applications. Int. J. Biol. Macromol..

[12] Huang F., Guo Y., Zhang R., Yi Y., Deng Y., Su D., Zhang M. (2014). Effects of drying methods on physicochemical and immunomodulatory properties of polysaccharide-protein complexes from litchi pulp. Molecules.

[13] Marra F., Zhang L., Lyng J.G. (2009). Radio frequency treatment of foods: Review of recent advances. J. Food Eng..

[14] Liu W., Li F., Wang P., Liu X., He J.-J., Xian M.-L., Zhao L., Qin W., Gan R.-Y., Wu D.-T. (2019). Effects of drying methods on the physicochemical characteristics and bioactivities of polyphenolic-protein-polysaccharide conjugates from *Hovenia dulcis*. Int. J. Biol. Macromol..

[15] Fu Y., Feng K.-L., Wei S.-Y., Xiang X.-R., Ding Y., Li H.-Y., Zhao L., Qin W., Gan R.-Y., Wu D.-T. (2020). Comparison of structural characteristics and bioactivities of polysaccharides from loquat leaves prepared by different drying techniques. Int. J. Biol. Macromol..

[16] Yuan Q., He Y., Xiang P.Y., Huang Y.J., Cao Z.W., Shen S.W., Zhao L., Zhang Q., Qin W., Wu D.T. (2020). Influences of different drying methods on the structural characteristics and multiple bioactivities of polysaccharides from okra (*Abelmoschus esculentus*). Int. J. Biol. Macromol..

[17] Guo C., Zhang N., Liu C., Xue J., Chu J., Yao X. (2020). Qualities and antioxidant activities of lotus leaf affected by different drying methods. Acta Physiol. Plant..

[18] Li F., Feng K.L., Yang J.C., He Y.S., Guo H., Wang S.P., Gan R.Y., Wu D.T. (2021). Polysaccharides from dandelion (*Taraxacum mongolicum*) leaves: Insights into innovative drying techniques on their structural characteristics and biological activities. Int. J. Biol. Macromol..

[19] Nie X.R., Li H.Y., Du G., Lin S., Hu R., Li H.Y., Zhao L., Zhang Q., Chen H., Wu D.T. (2019). Structural characteristics, rheological properties, and biological activities of polysaccharides from different cultivars of okra (*Abelmoschus esculentus*) collected in China. Int. J. Biol. Macromol..

[20] Yuan Q., He Y., Xiang P.Y., Wang S.P., Cao Z.W., Gou T., Shen M.M., Zhao L., Qin W., Gan R.Y. (2020). Effects of simulated saliva-gastrointestinal digestion on the physicochemical properties and bioactivities of okra polysaccharides. Carbohydr. Polym..

[21] Yuan Q., Lin S., Fu Y., Nie X.R., Liu W., Su Y., Han Q.H., Zhao L., Zhang Q., Lin D.R. (2019). Effects of extraction methods on the physicochemical characteristics and biological activities of polysaccharides from okra (*Abelmoschus esculentus*). Int. J. Biol. Macromol..

[22] Yan J.-K., Wu L.-X., Qiao Z.-R., Cai W.-D., Ma H. (2019). Effect of different drying methods on the product quality and bioactive polysaccharides of bitter gourd (*Momordica charantia* L.) slices. Food Chem..

[23] Zhu F. (2018). Interactions between cell wall polysaccharides and polyphenols. Crit. Rev. Food Sci. Nutr..

[24] Liu J., Wang X., Yong H., Kan J., Jin C. (2018). Recent advances in flavonoid-grafted polysaccharides: Synthesis, structural characterization, bioactivities and potential applications. Int. J. Biol. Macromol..

[25] Sun L., Wang C., Shi Q., Ma C. (2009). Preparation of different molecular weight polysaccharides from *Porphyridium cruentum* and their antioxidant activities. Int. J. Biol. Macromol..

[26] Yan S., Pan C., Yang X., Chen S., Qi B., Huang H. (2021). Degradation of codium cylindricum polysaccharides by H_2_O_2_-Vc-ultrasonic and H_2_O_2_-Fe^2+^-ultrasonic treatment: Structural characterization and antioxidant activity. Int. J. Biol. Macromol..

[27] Wang M., Zhao S., Zhu P., Nie C., Ma S., Wang N., Du X., Zhou Y. (2018). Purification, characterization and immunomodulatory activity of water extractable polysaccharides from the swollen culms of *Zizania latifolia*. Int. J. Biol. Macromol..

[28] Zhang W., Xiang Q., Zhao J., Mao G., Feng W., Chen Y., Li Q., Wu X., Yang L., Zhao T. (2020). Purification, structural elucidation and physicochemical properties of a polysaccharide from *Abelmoschus esculentus* L. (okra) flowers. Int. J. Biol. Macromol..

[29] Yang C., Gou Y., Chen J., An J., Chen W., Hu F. (2013). Structural characterization and antitumor activity of a pectic polysaccharide from *Codonopsis pilosula*. Carbohydr. Polym..

[30] Han K., Jin C., Chen H., Wang P., Yu M., Ding K. (2018). Structural characterization and anti-A549 lung cancer cells bioactivity of a polysaccharide from *Houttuynia cordata*. Int. J. Biol. Macromol..

[31] Yue H., Xu Q., Bian G., Guo Q., Fang Z., Wu W. (2020). Structure characterization and immunomodulatory activity of a new neutral polysaccharide SMP-0b from *Solanum muricatum*. Int. J. Biol. Macromol..

[32] Shakhmatov E.G., Toukach P.V., Michailowa C., Makarova E.N. (2014). Structural studies of arabinan-rich pectic polysaccharides from *Abies sibirica* L. Biological activity of pectins of *A. sibirica*. Carbohydr. Polym..

[33] Deng Y., Chen L.X., Han B.X., Wu D.T., Cheong K.L., Chen N.F., Zhao J., Li S.P. (2016). Qualitative and quantitative analysis of specific polysaccharides in *Dendrobium huoshanense* by using saccharide mapping and chromatographic methods. J. Pharm. Biomed. Anal..

[34] Morris V.J., Belshaw N.J., Waldron K.W., Maxwell E.G. (2013). The bioactivity of modified pectin fragments. Bioact. Carbohydr. Diet. Fibre.

[35] Guo Q., Ma Q., Xue Z., Gao X., Chen H. (2018). Studies on the binding characteristics of three polysaccharides with different molecular weight and flavonoids from corn silk (*Maydis stigma*). Carbohydr. Polym..

[36] Zhu R., Wang C., Zhang L., Wang Y., Chen G., Fan J., Jia Y., Yan F., Ning C. (2019). Pectin oligosaccharides from fruit of *Actinidia arguta*: Structure-activity relationship of prebiotic and antiglycation potentials. Carbohydr. Polym..

[37] Gao H., Wen J.J., Hu J.L., Nie Q.X., Chen H.H., Xiong T., Nie S.P., Xie M.Y. (2018). Polysaccharide from fermented *Momordica charantia* L. with *Lactobacillus plantarum* NCU116 ameliorates type 2 diabetes in rats. Carbohydr. Polym..

[38] Chen B., Long P., Sun Y., Meng Q., Liu X., Cui H., Lv Q., Zhang L. (2017). The chemical profiling of loquat leaf extract by HPLC-DAD-ESI-MS and its effects on hyperlipidemia and hyperglycemia in rats induced by a high-fat and fructose diet. Food Funct..

[39] Dou Z., Chen C., Fu X. (2019). The effect of ultrasound irradiation on the physicochemical properties and α-glucosidase inhibitory effect of blackberry fruit polysaccharide. Food Hydrocoll..

[40] Chen X.H., Chen G.J., Wang Z.R., Kan J.Q. (2020). A comparison of a polysaccharide extracted from ginger (*Zingiber officinale*) stems and leaves using different methods: Preparation, structure characteristics, and biological activities. Int. J. Biol. Macromol..

[41] Espinal-Ruiz M., Parada-Alfonso F., Restrepo-Sánchez L.P., Narváez-Cuenca C.E. (2014). Inhibition of digestive enzyme activities by pectic polysaccharides in model solutions. Bioact. Carbohydr. Diet. Fibre.

[42] Wu D.T., Liu W., Xian M.L., Du G., Liu X., He J.J., Wang P., Qin W., Zhao L. (2020). Polyphenolic-protein-polysaccharide complexes from *Hovenia dulcis*: Insights into extraction methods on their physicochemical properties and in vitro bioactivities. Foods.

